# Prevalence, antibiotic susceptibility, and presence of drug resistance genes in *Aeromonas* spp. isolated from freshwater fish in Kelantan and Terengganu states, Malaysia

**DOI:** 10.14202/vetworld.2021.2064-2072

**Published:** 2021-08-10

**Authors:** Nik Nur Fazlina Nik Mohd Fauzi, Ruhil Hayati Hamdan, Maizan Mohamed, Aziana Ismail, Ain Auzureen Mat Zin, Nora Faten Afifah Mohamad

**Affiliations:** Department of Paraclinical Studies, Faculty of Veterinary Medicine, Universiti Malaysia Kelantan, Pengkalan Chepa, 16100 Kota Bharu, Kelantan, Malaysia

**Keywords:** *Aeromonas*, antibiotic resistance genes, antibiotic susceptibility, freshwater fish

## Abstract

**Background and Aim:**

The emergence of antibiotic-resistant bacterial pathogens has been increasingly reported, which has resulted in a decreasing ability to treat bacterial infections. Therefore, this study investigated the presence of *Aeromonas* spp., including its antibiotic resistance in various fish samples, *Oreochromis* spp., *Clarias gariepinus*, and *Pangasius hypophthalmus*, obtained from Kelantan and Terengganu, Malaysia.

**Materials and Methods:**

In this study, 221 fish samples, of which 108 (*Oreochromis* spp., n=38; *C. gariepinus*, n=35; and *P. hypophthalmus*, n=35) were from Kelantan and 113 (*Oreochromis* spp., n=38; *C. gariepinus*, n=35; and *P. hypophthalmus*, n=40) were from Terengganu, were caught using cast nets. Then, samples from their kidneys were cultured on a Rimler Shott agar to isolate *Aeromonas* spp. Polymerase chain reaction (PCR) was used to confirm this isolation using specific gene primers for species identification. Subsequently, the isolates were tested for their sensitivity to 14 antibiotics using the Kirby–Bauer method, after which the PCR was conducted again to detect resistance genes: *sul1*, *strA*-*strB*, *aadA*, *bla*_TEM_, *bla*_SHV_, *tetA*-*tetE*, and *tetM*.

**Results:**

From the results, 61 isolates were identified as being from the genus *Aeromonas* using PCR, of which 28 were *Aeromonas jandaei*, 19 were *Aeromonas veronii*, seven were *Aeromonas hydrophila*, and seven were *Aeromonas sobria*. Moreover, 8, 12, and 8 of *A. jandaei*; 4, 3, and 12 of *A. veronii*; 6, 0, and 1 of *A. hydrophila*; and 3, 3, and 1 of *A. sobria* were obtained from *Oreochromis* spp., *C. gariepinus*, and *P. hypophthalmus*, respectively. In addition, the isolates showed the highest level of resistance to ampicillin (100%), followed by streptomycin (59.0%), each kanamycin and nalidixic acid (41.0%), neomycin (36.1%), tetracycline (19.7%), sulfamethoxazole (14.8%), and oxytetracycline (13.1%). Resistance to gentamicin and ciprofloxacin both had the same percentage (9.8%), whereas isolates showed the lowest resistance to norfloxacin (8.2%) and doxycycline (1.6%). Notably, all *Aeromonas* isolates were susceptible to chloramphenicol and nitrofurantoin. Results also revealed that the multiple antibiotic resistances index of the isolates ranged from 0.07 to 0.64, suggesting that the farmed fish in these areas were introduced to the logged antibiotics indiscriminately and constantly during their cultivation stages. Results also revealed that the *sul1* gene was detected in 19.7% of the *Aeromonas* isolates, whereas the tetracycline resistance genes, *tetA* and *tetE*, were detected in 27.9% and 4.9% of the isolates, respectively. However, β-lactam resistance genes, *bla*_TEM_ and *bla*_SHV_, were found in 44.3% and 13.1% of *Aeromonas* isolates, respectively, whereas *strA-strB* and *aadA* genes were found in 3.3% and 13.1% of the isolates, respectively.

**Conclusion:**

This study, therefore, calls for continuous surveillance of antibiotic-resistant *Aeromonas* spp. in cultured freshwater fish to aid disease management and better understand their implications to public health.

## Introduction

Aquaculture plays an important role in the food supply of Malaysia. Under the Economic Transformation Program, the Malaysian government established aquaculture as one of the key thrust areas for the agro-food industry [1]. In 2014, Malaysia’s annual per capita fish intake was one of the highest in Asia at 56.5 kg, with tilapia (*Oreochromis* spp.) and African catfish (*Clarias gariepinus*) being the favored farmed fish. Interestingly, in freshwater aquaculture, the African (*C. gariepinus*) and Pangasius (*Pangasius hypophthalmus*) catfishes being produced are leading because of a higher local demand, followed by tilapia (*Oreochromis* spp.), which is small in terms of production and was valued at RM223,000 (USD 58,000) [1].

Despite these interesting facts, bacterial infections are the most growing contagious concern in industrial fish farms and ornamental fish [2]. Studies have shown that captive fish are susceptible to many pathogenic bacteria that can cause kidney disease, dropsy, enteric redmouth, tuberculosis, vibriosis, motile aeromonad septicemia, bacterial gill infection, mouth fungus, tail and fin rot, and columnaris [3-7]. Furthermore, one of the most emerging bacteria that cause infectious diseases in freshwater aquaculture worldwide is *Aeromonas hydrophila* and other aeromonads [8,9]. These *Aeromonas* species can also cause motile aeromonads septicemia (MAS) in fish, with clinical symptoms, such as ulceration, ascitis, scale detachment, erosion, and exophthalmia being reported [10]. Apart from *A. hydrophila*, many disease-related aeromonads have been identified in tilapia as well, such as *Aeromonas sobria* [11], *Aeromonas dhakensis* (*A. hydrophila* subspecies dhakensis) [12], and *Aeromonas veronii* (synonyms of *Aeromonas ichthiosmia*, *Aeromonas culicicola*, and *Aeromonas allosaccharophila*) [13-16]. However, the occurrence of *A. hydrophila* infection was significantly higher in cultured fish than in wild species, such as Nile tilapia [17].

Antimicrobials have progressively been used in animal farming for disease prevention and treatment over the past few years, including as growth promoters [18]. However, their usage is based on modern medicine; the misuse of these antibiotics has increased the risk of emerging antimicrobial resistance cases in pathogenic and nonpathogenic bacteria. This has resulted in the lower treatment potency of commonly used antimicrobials in treating diseases, such as tuberculosis, pneumonia, and gastrointestinal infections, in humans [19]. In addition, during animal farming, antimicrobial deposits have been discovered in terrestrial, freshwater, and marine habitats close to agriculture and aquaculture facilities [20,21]. Antimicrobials are also applied in the feed or directly to water in aquaculture systems. Thus, they are proposed to subsequently be disposed into the environment by run-off water, sedimentation of feces, or uneaten feed pellets that can then be eaten by local fish or invertebrates [21-25]. The unconstrained use of antimicrobials in aquaculture can therefore transmit antibiotic-resistant bacteria, which are commonly transferred through R plasmids, with fish bacteria acting as intermediates [18,20,22,26-31].

Therefore, this study investigated the presence of *Aeromonas* spp., including its antibiotic resistance in various fish samples, *Oreochromis* spp., *Clarias gariepinus*, and *Pangasius hypophthalmus*, obtained from Kelantan and Terengganu, Malaysia.

## Materials and Methods

### Ethical approval

The study was approved by the Institutional Animal Care and Use Committee (IACUC), Faculty of Veterinary Medicine, University Malaysia Kelantan (UMK/FPV/ACUE/PG/4/2019).

### Study period and location

This study was conducted from February 2019 to December 2019. Samples were taken from three freshwater fish farms, each in state of Kelantan and Terengganu. In Kelantan, the farms located in Tumpat, Kota Bharu and Bachok. In Terengganu, two farms located in Kuala Terengganu and one farm in Hulu Terengganu. All the samples were processed *in situ* with an aseptic technique.

### Sample collection

Here, 221 freshwater fish were collected, with 108 samples from Kelantan and 113 samples from Terengganu. Of the 108 fish samples from Kelantan, 38 were red hybrid tilapia (*Oreochromis* spp.), 35 were African catfish (*C. gariepinus*), and the remaining 35 were Pangasius catfish (*P. hypophthalmus*). However, of the 113 fish samples collected from Terengganu, 38 were *Oreochromis* spp., 35 were *C. gariepinus*, and 40 were *P. hypophthalmus*. Next, a specimen of the kidneys was collected from these fish.

### Bacterial isolation and identification

The specimen was inoculated on Rimler Shott agar (RSA) (HiMedia, India) supplemented with novobiocin antibiotics and incubated at 30°C for 24 h. Next, yellow colonies on RSA were chosen and further sub-cultured on Trypticase soy agar (TSA) (Oxoid, Hampshire, UK) for purity. Subsequently, morphological and biochemical tests were used to identify all isolates, such as Gram staining, oxidase, catalase, and motility tests, after which the biochemical characteristics of *Aeromonas* spp. were examined using the analytical profile index 20E kit (bioMerieux, France) according to the manufacturer’s instructions. Finally, the strip was incubated at 30°C for 24 h.

### Confirmation of *Aeromonas* spp. using polymerase chain reaction (PCR) assay

Genomic DNA was extracted using the Bacterial Genomic DNA kit (Geneaid, USA) following the manufacturer’s instructions. To determine the presence of *Aeromonas* spp., a PCR assay was then conducted using 16S rRNA and a specific gene [32]. Next, PCR amplification was conducted using a Mastercycler gradient (Bio-Rad, USA). A final PCR volume of 25 μL containing 12.5 μL Go Taq^®^ Green Master Mix (Promega, USA), 1 μL of each 10 ρmol forward and reverse primers, and 2 μL DNA template were used. The conditions for thermocycling were set as follows: 94°C for 3 min, 35 cycles of 94°C for 60 s, 58°C for 60 s, 72°C for 1.5 min, and a final extension at 72°C for 3 min. Finally, amplified products were electrophoresed on 2.0% agarose gels, after which the gels were visualized and captured using GelDoc (Bio-Rad).

### Determination of antibiotic susceptibility and multiple antibiotic resistance (MAR) index of selected bacteria

The isolates were tested for sensitivity to 14 antibiotics: Ampicillin (10 μg), gentamicin (10 μg), neomycin (30 μg), streptomycin (10 μg), kanamycin (30 μg), tetracycline (30 μg), oxytetracycline (30 μg), ciprofloxacin (5 μg), norfloxacin (10 μg), nalidixic acid (30 μg), chloramphenicol (30 μg), sulfamethoxazole (25 μg), doxycycline (30 μg), and nitrofurantoin (300 μg). Kirby–Bauer’s disc diffusion method was then used to assess the patterns of antibiotic sensitivity of the isolates. Inhibition zone results were subsequently interpreted as sensitive (S), intermediate (I), and resistant (R) according to the reference standard by the Clinical and Laboratory Standard Institute [33].

MAR index was calculated using the formula provided by Sarter *et al*. [34]:

X/(Y×Z)

Where, X=Total cases of antibiotic resistance; Y=Total number of isolates; Z=Total number of isolates

The MAR index value of equal to, or less than, 0.2 was defined as antibiotics that were seldom or never used.

### Detection of associated drug resistance genes

Resistance genes were detected using PCR amplification with the different primers as described in Table-1 [35-41]. Assays were then conducted in 25 μL volume mixtures, according to the manufacturer’s protocol (Promega, USA). Next, all PCR reactions were subjected to amplification according to the cycling parameter suggested by a previous researcher (Table-1). Finally, PCR products were run on 2.0% agarose, after which the gel was visualized and captured using Gel Doc (Bio-Rad).

**Table-1:** List of primers used for detection of antibiotic resistance genes.

Primer	Nucleotide sequence (5’–3’)	Product size (bp)	References
sul1-F	CTTCGATGAGACCCGGCGGC	436	[35]
sul1-R	GCAAGGCGGAAACCCGCGCC		
aadA-F	GAGAACATAGCGTTGCCTTGGTCG	198	[36]
aadA-R	GCGCGATTTTGCCGGTTA		
strA-strB-F	TTGAATCGAACTAATAT	1640	[37]
strA-strB-R	CTAGTATGACGTCTGTCG		
blaTEM-F	ATGAGTATTCAACATTTCCG	867	[38]
blaTEM-R	CTGACAGTTACCAATGCTTA		
blaSHV-F	GGTTATGCGTTATATTCGCC	867	[38]
blaSHV-R	TTAGCTTTGCCAGTGCTC		
tetA-F	GTAATTCTGAGCACTGTCGC	956	[39,40]
tetA-R	CTGCCTGGACAACATTGCTT		
tetB-F	CTCAGTATTCCAAGCCTTTG	535	[39,40]
tetB-R	CTAAGCACTTGTCTCCTGTT		
tetC-F	TCTAACAATGCGCTCATCGT	588	[39,40]
tetC-R	GGTTGAAGGCTCTCAAGGGC		
tetD-F	ATTACACTGCTGGACGCGAT	1070	[39,40]
tetD-R	CTGATCAGCAGACAGATTGC		
tetE-F	GTGATGATGGCACTGGTCAT	1198	[39,40]
tetE-R	CTCTGCTGTACATCGCTCTT		
tetM-F	GTTAAATAGTGTTCTTGGAG	650	[41]
tetM-R	CTAAGATATGGCTCTAACAA		

## Results

From the results, 61 isolates obtained from freshwater fish samples were identified as genus *Aeromonas* using PCR. Table-2 shows that from the 61 *Aeromonas* spp. isolated, 22 isolates were from *P. hypophthalmus*, 19 from *Oreochromis* spp., and 20 from *C. gariepinus*. Furthermore, *Aeromonas* species isolated from freshwater fish in Kelantan were higher (43 isolates) than those from Terengganu (18 isolates).

**Table-2:** Prevalence of *Aeromonas* spp. isolated from freshwater fish.

Host species	*Aeromonas* spp. isolated (n)	Kelantan (n, %)	Terengganu (n, %)
*Pangasius hypophthalmus*	22	20 (90.9)	2 (9.1)
*Oreochromis* spp.	19	9 (47.4)	10 (52.6)
*Clarias gariepinus*	20	14 (70.0)	6 (30.0)
Total	61	43 (70.5)	18 (29.5)

**Figure-1:**
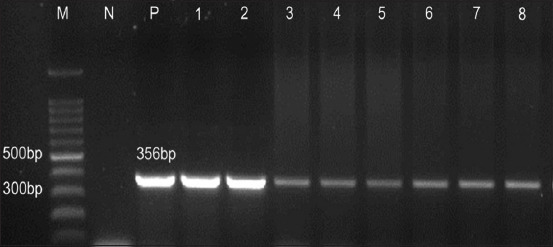
Representative of polymerase chain reaction (PCR) positives for 16S rRNA of genus *Aeromonas*. Lane M: 1 Kbp DNA marker (Promega, USA); Lane N: negative control; Lane P: positive control; Lanes 1-8: Positive *Aeromonas* with 356 bp PCR products.

**Figure-2:**
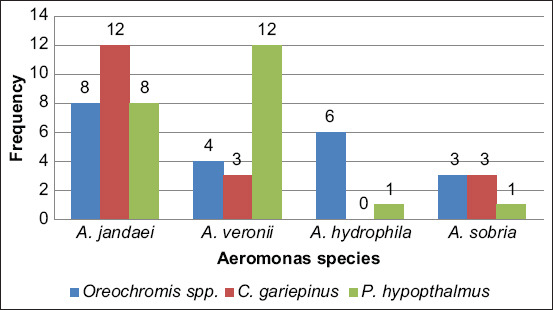
Distribution of *Aeromonas* species isolated from freshwater fish.

Figure-1 shows the confirmed identification using the PCR assay of *Aeromonas* spp. The positive isolates for the 16S rRNA gene were then sent for sequencing. Figure-2 shows the distribution of *Aeromonas* species according to each state in Kelantan and Terengganu. Four types of *Aeromonas* species were obtained during this study, with 28 isolates of *Aeromonas jandaei*, 19 isolates of *A. veronii*, seven isolates of *A. hydrophila*, and seven isolates of *A. sobria*. Furthermore, *Aeromonas jandei* and *A. veronii* were detected in both samples from Kelantan and Terengganu, whereas *A. hydrophila* and *A. sobria* were detected only in samples from Kelantan.

**Figure-3:**
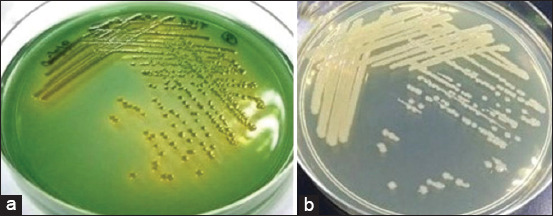
*Aeromonas veronii* on; (a) Rimler Shott agar; (b) Trypticase Soy agar.

Figure-3 shows *Aeromonas* spp. colonies formed on TSA, which were creamy in color, round, and convex, whereas *Aeromonas* colonies on RSA were yellow-green in color, round, and convex. The biochemical test results from *Aeromonas* spp. isolates revealed Gram-negative staining, rod-shaped, motile, fermentative, oxidase-positive, catalase-positive, and indole negative characteristics.

**Figure-4:**
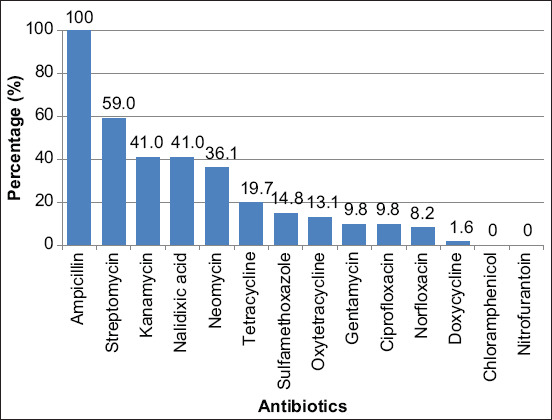
Antibiotic resistant of *Aeromonas* isolates.

In addition, all *Aeromonas* isolates displayed varying trends of resistance, where all isolates were ampicillin-resistant (100%), followed by streptomycin (59.0%), kanamycin and nalidixic acid with the same percentage (41.0%), neomycin (36.1%), tetracycline (19.7%), sulfamethoxazole (14.8%), and oxytetracycline (13.1%). Gentamicin and ciprofloxacin both had the same percentage resistance (9.8%), whereas norfloxacin (8.2%) and doxycycline (1.6%) had the lowest (Figure-4). However, all *Aeromonas* isolates were sensitive to chloramphenicol and nitrofurantoin.

**Figure-5:**
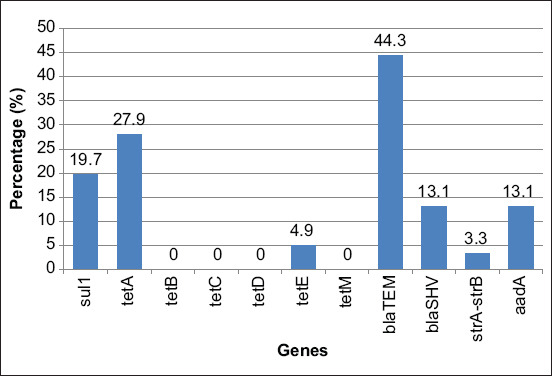
The presence of antibiotic resistance genes in *Aeromonas* isolated from freshwater fish.

Figure-5 shows the presence of antibiotic resistance genes in *Aeromonas* isolates. Results showed that the *sul1* gene (related to sulfonamide resistance) was detected in 19.7% of the *Aeromonas* isolates. However, for tetracycline resistance genes, only *tetA* and *tetE* were detected in 27.9% and 4.9% of isolates, respectively. In addition, the β-lactam resistance genes, *bla*_TEM_ and *bla*_SHV_, were found in 44.3% and 13.1% of *Aeromonas* isolates, respectively, whereas the *strA-strB* gene (related to streptomycin resistance) was found in 3.3% of the isolates, and the *aadA* gene (related to streptomycin and spectinomycin resistance) in 13.1% of the isolates. Table-3 shows the resistance phenotype and antibiotic resistance genes of all *Aeromonas* spp. isolates.

**Table-3:** Resistance phenotype and presence of antibiotic resistance genes in *Aeromonas* spp. isolated from freshwater fish.

No.	Isolates	Identification	Fish species	Location	Resistance phenotype	Genes detected by PCR	MAR Index
1.	K1K2	*A. sobria*	*C. gariepinus*	Kelantan	Amp-N-S-K	bla*_TEM_*, bla*_SHV_*	0.29
2.	K1K3	*A. sobria*	*C. gariepinus*	Kelantan	Amp-N-S-K-Na-Sxt	sul1, tetA	0.43
3.	K2K11	*A. jandaei*	*C. gariepinus*	Kelantan	Amp-S-Na-Sxt-Ot	-	0.36
4.	K2K12	*A. sobria*	*C. gariepinus*	Kelantan	Amp-N-S-K-Te-Cip-Na-Ot	tetA	0.57
5.	K2K15	*A. jandaei*	*C. gariepinus*	Kelantan	Amp-N-S-K-Na	bla*_TEM_*	0.36
6.	K2K16	*A. veronii*	*C. gariepinus*	Kelantan	Amp-N-S-K-Na-Sxt-Ot	sul1, tetE, bla*_TEM_*	0.50
7.	K3K22	*A. veronii*	*C. gariepinus*	Kelantan	Amp-Te-Na-Ot	bla*_TEM_*	0.29
8.	K3K24	*A. jandaei*	*C. gariepinus*	Kelantan	Amp-N-S	bla*_TEM_*	0.21
9.	K3K25	*A. veronii*	*C. gariepinus*	Kelantan	Amp-Te-Na-Sxt-Ot	sul1, tetA, bla*_TEM_*	0.36
10.	K3K26	*A. jandaei*	*C. gariepinus*	Kelantan	Amp-S	bla*_TEM_*	0.14
11.	K3K27	*A. jandaei*	*C. gariepinus*	Kelantan	Amp	bla*_TEM_*	0.07
12.	K3K28	*A. jandaei*	*C. gariepinus*	Kelantan	Amp	-	0.07
13.	K3K29	*A. jandaei*	*C. gariepinus*	Kelantan	Amp-N-S-K-Na-Ot	*tetA, strA-strB, bla_TEM_*	0.43
14.	K3K30	*A. jandaei*	*C. gariepinus*	Kelantan	Amp-S-Na	*tetA, bla_TEM_*	0.21
15.	K1P2	*A. sobria*	*P. hypopthalmus*	Kelantan	Amp-N-S-K-Te-Na	*tetA, strA-strB, aadA*	0.43
16.	K1P5	*A. veronii*	*P. hypopthalmus*	Kelantan	Amp	-	0.07
17.	K2P1	*A. hydrophila*	*P. hypopthalmus*	Kelantan	Amp	-	0.07
18.	K2P2	*A. jandaei*	*P. hypopthalmus*	Kelantan	Amp-Te	*tetE*	0.14
19.	K2P3	*A. jandaei*	*P. hypopthalmus*	Kelantan	Amp-Na	-	0.14
20.	K2P4	*A. veronii*	*P. hypopthalmus*	Kelantan	Amp-S	-	0.14
21.	K2P5	*A. veronii*	*P. hypopthalmus*	Kelantan	Amp-S-K	*bla_TEM_*	0.21
22.	K2P6 (a)	*A. veronii*	*P. hypopthalmus*	Kelantan	Amp	-	0.07
23.	K2P6 (b)	*A. veronii*	*P. hypopthalmus*	Kelantan	Amp-N-K	*bla_TEM_*	0.21
24.	K2P7	*A. jandaei*	*P. hypopthalmus*	Kelantan	Amp-S	-	0.14
25.	K2P8 (a)	*A. jandaei*	*P. hypopthalmus*	Kelantan	Amp-S-K	*bla_TEM_*	0.21
26.	K2P8 (b)	*A. jandaei*	*P. hypopthalmus*	Kelantan	Amp-N-S-K-Na	*bla_TEM_, bla_SHV_*	0.36
27.	K2P10	*A. veronii*	*P. hypopthalmus*	Kelantan	Amp-S-K	-	0.21
28.	K3P4	*A. jandaei*	*P. hypopthalmus*	Kelantan	Amp-S-K	-	0.21
29.	K3P5 (a)	*A. veronii*	*P. hypopthalmus*	Kelantan	Amp-S-K	-	0.21
30	K3P5 (b)	*A. veronii*	*P. hypopthalmus*	Kelantan	Amp-S-K	*bla_TEM_*	0.21
31.	K3P6 (a)	*A. veronii*	*P. hypopthalmus*	Kelantan	Amp-N-S-K	*bla_TEM_, bla_SHV_*	0.29
32.	K3P6 (b)	*A. veronii*	*P. hypopthalmus*	Kelantan	Amp-S-K	-	0.21
33.	K3P9 (a)	*A. veronii*	*P. hypopthalmus*	Kelantan	Amp-S-K	*bla_TEM_*	0.21
34.	K3P9 (b)	*A. veronii*	*P. hypopthalmus*	Kelantan	Amp-S-K	-	0.21
35.	K1T2 (a)	*A. hydrophila*	Oreochromis spp.	Kelantan	Amp-Cn-N-S-K-Cip-Nor-Na-Sxt	*sul1, tetA, bla_TEM_, bla_SHV_, aadA*	0.64
36.	K1T2 (b)	*A. sobria*	*Oreochromis* spp.	Kelantan	Amp-N-S-K-Te-Na-Sxt	*sul1, tetA, bla_TEM_*	0.50
37.	K2T3 (a)	*A. sobria*	*Oreochromis* spp.	Kelantan	Amp-Cn-N-S-K-Cip-Nor-Na-Sxt	*sul1, tetA, bla_TEM_, bla_SHV_, aadA*	0.64
38.	K2T3 (b)	*A. sobria*	*Oreochromis* spp.	Kelantan	Amp-N-S-Te-Na	*tetA, bla_TEM_, aadA*	0.36
39.	K2T6 (a)	*A. hydrophila*	*Oreochromis* spp.	Kelantan	Amp-N-S-Te-Na	*tetA, bla_TEM_, aadA*	0.36
40.	K2T6 (b)	*A. hydrophila*	*Oreochromis* spp.	Kelantan	Amp-Cn-N-S-K-Na-Sxt	*sul1, tetA, bla_TEM_*	0.50
41.	K3T8	*A. hydrophila*	*Oreochromis* spp.	Kelantan	Amp-Cn-N-S-K-Te-Cip-Nor-Na	*tetA, bla_TEM_, bla_SHV_*	0.64
42.	K3T10	*A. hydrophila*	*Oreochromis* spp.	Kelantan	Amp-Cn-N-S-K-Te-Cip-Nor-Na	*tetA, bla_TEM_, bla_SHV_, aadA*	0.64
43.	K3T11	*A. hydrophila*	*Oreochromis* spp.	Kelantan	Amp-Cn-N-S-Cip-Nor-Na-Sxt	*sul1, tetA, bla_TEM_, bla_SHV_, aadA*	0.57
44.	T2K5	*A. jandaei*	*C. gariepinus*	Terengganu	Amp-Na	*tetE*	0.14
45.	T2K4	*A. jandaei*	*C. gariepinus*	Terengganu	Amp	-	0.07
46.	T3K6	*A. jandaei*	*C. gariepinus*	Terengganu	Amp-Te-Na-Do-Ot	*tetA*	0.36
47.	T3K8	*A. jandaei*	*C. gariepinus*	Terengganu	Amp	-	0.07
48.	T1T7	*A. jandaei*	*Oreochromis* spp.	Terengganu	Amp	*sul1*	0.07
49.	T1T10 (b)	*A. veronii*	*Oreochromis* spp.	Terengganu	Amp	*sul1, aadA*	0.07
50.	T1T4 (a)	*A. jandaei*	*Oreochromis* spp.	Terengganu	Amp	*sul1*	0.07
51.	T1K6	*A. veronii*	*Oreochromis* spp.	Terengganu	Amp-N-Te-Sxt-Ot	*sul1, tetA*	0.36
52.	T1K7	*A. veronii*	*Oreochromis* spp.	Terengganu	Amp-S	-	0.14
53.	T1T6	*A. jandaei*	*Oreochromis* spp.	Terengganu	Amp-Na-S	-	0.21
54.	T1T9	*A. veronii*	*Oreochromis* spp.	Terengganu	Amp	-	0.07
55.	T2T1	*A. jandaei*	*Oreochromis* spp.	Terengganu	Amp	-	0.07
56.	T2T3	*A. jandaei*	*Oreochromis* spp.	Terengganu	Amp	*bla_TEM_*	0.07
57.	T2T5 (a)	*A. jandaei*	*Oreochromis* spp.	Terengganu	Amp	-	0.07
58.	T2T6	*A. jandaei*	*Oreochromis* spp.	Terengganu	Amp-S	-	0.14
59.	T2T7	*A. jandaei*	*Oreochromis* spp.	Terengganu	Amp-Na-S	-	0.21
60.	T1P8	*A. jandaei*	*P. hypopthalmus*	Terengganu	Amp	-	0.07
61.	T2P3	*A. jandaei*	*P. hypopthalmus*	Terengganu	Amp	-	0.07

Amp=Ampicillin (10 μg), Cn=Gentamicin (10 μg), N=Neomycin (30 μg), S=Streptomycin (10 μg), K=Kanamycin (30 μg), Te=Tetracycline (30 μg), Cip=Ciprofloxacin (5 μg), Nor=Norfloxacin (10 μg), Na=Nalidixic acid (30 μg), Sxt=Sulfamethoxazole (25 μg), C=Chloramphenicol (30 μg), Do=Doxycycline (30 μg), F=Nitrofurantoin (300 μg), Ot=Oxytetracycline (30 μg). MAR=Multiple antibiotic resistance, PCR=Polymerase chain reaction, *A. sobria=Aeromonas sobria, C. gariepinus=Clarias gariepinus, A. jandaei=Aeromonas jandaei, A. veronii=Aeromonas veronii,*

*P. hypopthalmus=Pangasiusr hypopthalmus, A. hydrophila=Aeromonas hydrophila*

## Discussion

H_2_S production is one of the features of *Aeromonas* spp. pathogenic piscine strains [42]. Shotts and Rimler [43] indicated that *Aeromonas* spp. grown on an RSA medium formed yellow colonies; however, the colonies with black centers had to be tested for oxidase production to exclude the probability of *Citrobacter* spp. or other species of bacteria. MAS is broad, which includes *A. hydrophila* and several species of *Aeromonas* that have been reported to be risks to freshwater fish in aquaculture [13,14,44,45]. Motile aeromonad infections are possibly the most significant bacterial infection in freshwater fish. They are also discovered regularly in the intestines and gills of freshwater fish. Therefore, in this study, bacteria of the genus *Aeromonas* were isolated from the kidneys of red hybrid tilapia (*Oreochromis* spp.), including African (*C. gariepinus*) and Pangasius (*P. hypophthalmus*) catfishes obtained from the states of Kelantan and Terengganu in Malaysia.

Furthermore, among the 61 isolates from the genus *Aeromonas* isolated, 28 isolates were *A. jandaei*, 19 were *A. veronii*, and seven isolates were *A. hydrophila* and *A. sobria*, respectively. These results are in agreement with those observed in earlier studies by Radu *et al*. [46] that found *A. veronii*, *A. sobria*, *A. hydrophila*, and *A. caviae* in the market fish samples from Selangor state in Malaysia. In India, *A. hydrophila* has also been isolated from fish obtained from retail shops [47]. In addition, Ashiru *et al*. [48] isolated *A. hydrophila*, *A. caviae*, and *A. sobria* in catfish (*Clarias betrachus*) and tilapia fish (*Tilapia nilotica*) obtained from the Makoko market in Nigeria. The authors reported that *A. caviae* was predominant in tilapia fish, whereas *A. hydrophila* and *A. sobria* were predominant in catfish. Other studies have also reported that *A. jandaei* is pathogenic to aquaculture fish, such as European eels (*Anguilla anguilla*) [49] and Pangasius catfish (*P. hypophthalmus*) [50]. Besides, other studies have shown that *A. veronii* infected high numbers of fish, such as Chinese long snout catfish (*Leiocassis longirostris*) [51], loach (*Misgurnus anguillicaudatus*) [45], Oscar (*Astronotus ocellatus*) [52], and tilapia (*Oreochromis* spp.) [13,14,44]. This bacterial genus attacks catfish, which is among the main freshwater resources, and infects all sizes of fish as well, which can lead to death and result in big losses of freshwater fish production [53].

*Aeromonas* genus generates single or MARs rapidly, indicating that this genus is an effective marker of antimicrobial resistance in the freshwater aquaculture system [54]. The MAR index varying from 0.07 to 0.64 with 60% (37/61) of the isolates have MAR indices of more than 0.2 (high-risk source of contamination), suggesting that the *Aeromonas* spp. in these farmed fish have been exposed to a high level of antibiotics during the cultivation processes, which can result in the development of antibiotic resistance among the bacteria isolates. Results from this study prove this fact, which revealed a high level of multi-drug resistance (MDR) among the isolates tested (Table-3). However, the percentage of MAR index of more than 0.2 in this study (60%) was much lower than that obtained from the study by Odeyemi and Ahmad [55] in *Aeromonas* spp., isolated from 53 aquatic samples in Melaka, Malaysia (100%). This result indicates that the indiscriminate use of antibiotics in West Coast Malaysia (Melaka) is higher than in East Coast Malaysia (Kelantan and Terengganu). High resistance of MDR due to *Aeromonas* spp. has also been reported as serious public health pathogens that cause gastroenteritis, septicemia, and skin infections in humans, which enter the human body through contaminated food and water consumption, including skin lesions [56].

In this study, all *Aeromonas* isolates were highly ampicillin-resistant. A previous study reported that these *Aeromonas* species acquired β-lactams resistance through the expression of chromosomal lactamases [57]. This finding is also proposed to be due to a high intrinsic β-lactam resistance, which is enhanced by an active efflux mechanism or cooperation through external membrane impermeability or secondary resistance mechanisms known as β-lactamases or antibiotic efflux pumps [54,57,58]. Furthermore, resistance rates to tetracycline, oxytetracycline, streptomycin, kanamycin, nalidixic acid, neomycin, sulfamethoxazole, ciprofloxacin, and gentamicin have also been recorded, which is suggested to be due to the extensive consumption of such antimicrobials in the ornamental fishery [59,60]. All isolates were also susceptible to chloramphenicol and nitrofurantoin. This observation is due to that these antibiotics were banned in Malaysia for use in treating aquatic animal diseases [61]. Several antibiotics were completely banned as well for food animals and aquaculture in Malaysia because of serious toxicity and the development of antibiotic-resistant bacterial strains, such as avoparcin, chloramphenicol, nitrofurans (i.e., nitrofurantoin, nitrofurazone, furazolidone, and furaltadone), teicoplanin, vancomycin, and norfloxacin [61,62].

Furthermore, in this study, no trends of significant antibiotic resistance specific to the fish species were observed. The current findings follow other research on MDR occurrence from aquatic habitats and seafood samples in *Aeromonas* spp. [63,64]. These classes of antibiotics are broadly used worldwide as well, particularly in developing countries in Asia, because of their low cost and diverse-spectrum antimicrobial activity, which increases the chances for any bacterial species to develop resistance to these antibiotics [65,66].

The presence of resistance genes was also detected in several of the isolates during this study, including those encoding resistance to ampicillin (*bla*_TEM_ and *bla*_SHV_), streptomycin (*aadA* and *strA-strB*), and tetracyclines (*tetA* and *tetE*). The present findings agree with earlier studies where *tetA* genes were the most significant determinants of tetracycline resistance and have commonly been observed in *Aeromonas* spp. [39,67]. Increased resistance to β-lactam antimicrobials (penicillins and derivatives, cephalosporins, carbapenems, and monobactams) through the existence of genes that code for the formation of β-lactamase has also been reported [68]. In addition, Jones-Dias *et al*. [69] mentioned that in *Aeromonas* spp., three main β-lactamases were identified: Metallo-β-lactamase Class B, cephalosporinase Class C, and penicillinase Class D. Fosse *et al*. [70] have also categorized the β-lactamases into five (i*v) groups of *Aeromonas* species: (i) The *A. hydrophila* complex strains exhibiting Classes B, C, and D β-lactamases; (ii) the *A. caviae* strains exhibiting Classes C and D β-lactamases; (iii) the *A. veronii* strains identifying Classes B and D lactamases; (iv) the *Allium schubertii* strains recognizing Class D lactamases; and (v) the *Aeromonas trota* strains containing Class C β-lactamases. It is also suggested that several isolates of *A. veronii* biovar sobria do contain a class C cephalosporinase [58]. Therefore, because of the presence of β-lactamase genes, increased resistance to β-lactam antibiotics was reported in the *Aeromonas* genus [4,68,71,72].

## Conclusion

This study has identified several *Aeromonas* spp. that are resistant to several types of antibiotics in freshwater fish from Kelantan and Terengganu states, with 60% (37/61) of the isolates having a MAR index of more than 0.2. The result suggests that aquaculture waste deposited into the aquatic ecosystems is one of the factors that enhance the incidence of aeromonad MDR in the river water. The presence of *Aeromonas* species in freshwater fish can thus be a major problem if the fish is not cooked properly. This drug resistance has become a major public health concern since these fish species (*Oreochromis* spp., *C. gariepinus*, and *P. hypophthalmus*) are important sources of aquatic proteins consumed in Malaysia. Therefore, regular surveillance for antibiotic resistance of *Aeromonas* spp. should be conducted among freshwater fish. Finally, more intensive studies should discover the specific existence of antibiotic resistance in *Aeromonas* spp., including the levels of antibiotics that affect their resistance profile.

## Authors’ Contributions

NNFNMF: Designed the study and drafted the manuscript. MM, RHH, AI, AAMZ, and NFAM: Data analysis. RHH and MM: Direction and supervision of the study. All authors read and approved the final manuscript.

## References

[1] (2015). DoF (Department of Fisheries).

[2] Sousa H, Hinzmann M (2020). Review:Antibacterial components of the Bivalve's immune system and the potential of freshwater bivalves as a source of new antibacterial compounds. Fish Shell. Immunol.

[3] Aberoum A, Jooyandeh H (2010). A review on occurrence and characterization of the *Aeromonas* species from marine fishes. World J. Fish Mar. Sci..

[4] Carvalho M.J, Martínez-Murcia A, Esteves A.C, Correia A, Saavedra M.J (2012). Phylogenetic diversity, antibiotic resistance and virulence traits of *Aeromonas* spp. from untreated waters for human consumption. Int. J. Food Microbiol..

[5] Ghenghesh K.S, Ahmed S.F, El-Khalek R.A, Al-Gendy A, Klena J (2008). *Aeromonas*-associated infections in developing countries. J. Infect. Dev. Ctries..

[6] Lee S.W, Wee W (2012). Characterization of *Vibrio alginolyticus* isolated from white leg shrimp (*Litopenaeus vannamei*) with emphasis on its antibiogram and heavy metal resistance pattern. Vet. Arh..

[7] Abbott S.L, Cheung W.K.W, Janda J.M (2003). The genus *Aeromonas*:Biochemical characteristics, atypical reactions, and phenotypic identification schemes. J. Clin. Microbiol..

[8] Dong H.T, Techatanakitarnan C, Jindakittikul P, Thaiprayoon A, Taengphu S, Charoensapsri W, Khunrae P, Rattanarojpong T, Senapin S (2017). *Aeromonas jandaei* and *Aeromonas veronii* caused disease and mortality in Nile tilapia, *Oreochromis niloticus* (L.). J. Fish Dis..

[9] Hassan M.A, Noureldin E.A, Mahmoud M.A, Fita N.A Molecular identification and epizootiology of *Aeromonas veronii* infection among farmed *Oreochromis niloticus* in Eastern Province, KSA. Egypt. J. Aquat. Res..

[10] Stratev D, Odeyemi O.A (2017). An overview of motile *Aeromonas* septicaemia management. Aquac. Int.

[11] Li Y, Cai S.H (2011). Identification and pathogenicity of *Aeromonas*
*sobria* on Tail-rot disease in Juvenile Tilapia *Oreochromis niloticus*. Curr. Microbiol..

[12] Soto-Rodriguez S.A, Cabanillas-Ramos J, Alcaraz U, Gomez-Gil B, Romalde J.L (2013). Identification and virulence of *Aeromonas dhakensis*, *Pseudomonas mosselii* and *Microbacterium paraoxydans* isolated from Nile tilapia, *Oreochromis niloticus*, cultivated in Mexico. J. Appl. Microbiol..

[13] Dong H.T, Rodkhum C, Le H.D, Sangsuriya P, Senapin S, Jitrakorn S, Jitrakorn S, Saksmerprome V, Nguyen V.V (2015). Naturally concurrent infections of bacterial and viral pathogens in disease outbreaks in cultured Nile tilapia (*Oreochromis niloticus*) farms. Aquaculture.

[14] Eissa I.A.M, El-Lamei M, Sherif M, Youssef F, Zaki M.S, Bakry M (2015). Detection of hemolysin gene and antibiogram of *Aeromonas veronii* biovar sobria isolated from mass mortalities in cultured Nile Tilapia in El-Sharkia governorate, Egypt,”. Life Sci. J.

[15] Huys G, Cnockaert M, Swings J (2005). *Aeromonas culicicola* Pidiyar *et al*. 2002 is a later subjective synonym of *Aeromonas veronii* Hickman-Brenner *et al*. 1987. Syst. Appl. Microbiol..

[16] Nhung P.H, Hata H, Ohkusu K, Noda M, Shah M.M, Goto K, Ezaki T (2007). Use of the novel phylogenetic marker dnaJ and DNA-DNA hybridization to clarify interrelationships within the genus *Aeromonas*. Int. J. Syst. Evol. Microbiol..

[17] Ibrahem M.D, Mostafa M.M, Arab R.M.H, Rezk M.A (2008). Prevalence of *Aeromonas hydrophila* Infection in Wild and Cultured Tilapia Nilotica (*O. niloticus*) in. 8^th^ International Symposium on Tilapia In Aquaculture.

[18] Van Boeckel T.P, Brower C, Gilbert M, Grenfell B.T, Levin S.A, Robinson T.P, Teillant A, Laxminarayan R (2015). Global trends in antimicrobial use in food animals. Proc. Natl. Acad. Sci. U. S. A..

[19] Fair R.J, Tor Y (2014). Antibiotics and bacterial resistance in the 21^st^ century. Perspect. Medicin. Chem..

[20] Nakayama T, Hoa T.T.T, Harada K, Warisaya M, Asayama M, Hinenoya A, Lee J.W, Phu T.M, Ueda S, Sumimura Y, Hirata K, Phuong N.T, Yamamoto Y (2017). Water metagenomic analysis reveals low bacterial diversity and the presence of antimicrobial residues and resistance genes in a river containing wastewater from backyard aquacultures in the Mekong Delta, Vietnam. Environ. Pollut..

[21] Rico A, Van den Brink P.J (2014). Probabilistic risk assessment of veterinary medicines applied to four major aquaculture species produced in Asia. Sci. Total Environ..

[22] Andrieu M, Rico A, Phu T.M, Huong D.T.T, Phuong N.T, Van den Brink P.J (2015). Ecological risk assessment of the antibiotic enrofloxacin applied to Pangasius catfish farms in the Mekong Delta, Vietnam. Chemosphere.

[23] Muziasari W.I, Pitkänen L.K, Sørum H, Stedtfeld R.D, Tiedje J.M, Virta M (2017). The resistome of farmed fish feces contributes to the enrichment of antibiotic resistance genes in sediments below baltic sea fish farms. Front. Microbiol..

[24] Rico A, Phu T.M, Satapornvanit K, Min J, Shahabuddin A.M, Henriksson P.J.G, Murray F.J, Little D.C, Dalsgaard A, Van den Brinka P.J (201). Use of veterinary medicines, feed additives and probiotics in four major internationally traded aquaculture species farmed in Asia. Aquaculture.

[25] Buschmann A.H, Tomova A, Lopez A, Maldonado M.A, Henriquez L.A, Ivanova L, Moy F, Godfrey H.P, Cabello F.C (2012). Salmon aquaculture and antimicrobial resistance in the marine environment. PLoS One.

[26] Hatosy S.M, Martiny A.C (2015). The ocean as a global reservoir of antibiotic resistance genes. Appl. Environ. Microbiol..

[27] Rico A, Oliveira R, McDonough S, Matser A, Khatikarn J, Satapornvanit K (2014). Use, fate and ecological risks of antibiotics applied in tilapia cage farming in Thailand. Environ. Pollut..

[28] Kümmerer K (2009). Antibiotics in the aquatic environment-a review-Part I. Chemosphere.

[29] Baquero F, Martínez J.L, Cantón R (2008). Antibiotics and antibiotic resistance in water environments. Curr. Opin. Biotechnol.

[30] Robinson T.P, Bu D.P, Carrique-Mas J, Fevre E.M, Gilbert M, Grace D, Hay S.I, Jiwakanon J, Kakkar M, Kariuki S, Laxminarayan R, Lubroth J, Magnusson U, Thi Ngoc P, Van Boeckel T.P, Woolhouse M.E.J (2016). Antibiotic resistance is the quintessential one health issue. Trans. R. Soc. Trop. Med. Hyg.

[31] Marshall B.M, Levy S.B (2011). Food animals and antimicrobials:Impacts on human health. Clin. Microbiol. Rev.

[32] Hussain I.A, Jeyasekaran G, Shakila R.J, Raj K.T, Jeevithan E (2014). Detection of hemolytic strains of *Aeromonas hydrophila* and *A. sobria* along with other *Aeromonas* spp. from fish and fishery products by multiplex PCR. J. Food Sci. Technol..

[33] CLSI (2018). Performance Standards for Antimicrobial Susceptibility Testing, CLSI Supplement M100.

[34] Sarter S, Kha Nguyen H.N, Hung L.T, Lazard J, Montet D (2007). Antibiotic resistance in Gram-negative bacteria isolated from farmed catfish. Food Control.

[35] Sundström L, Rådström P, Swedberg G, Sköld O (1988). Site-specific recombination promotes linkage between trimethoprim-and sulfonamide resistance genes. Sequence characterization of *dhfrV* and *sulI* and a recombination active locus of Tn 21. MGG Mol. Gen. Genet..

[36] Sunde M, Norström M (2005). The genetic background for streptomycin resistance in *Escherichia*
*coli* influences the distribution of MICs. J. Antimicrob. Chemother..

[37] Han H.S, Koh Y.J, Hur J.S, Jung J.S (2004). Occurrence of the *strA-strB* streptomycin resistance genes in *Pseudomonas* species isolated from kiwifruit plants. J. Microbiol..

[38] Rasheed J.K, Jay C, Metchock B, Berkowitz F, Weigel L, Crellin J (1997). Evolution of extended-spectrum b-lactam resistance (SHV-8) in a strain of *Escherichia coli* during multiple episodes of bacteremia. Antimicrob. Agents Chemother..

[39] Schmidt A.S, Bruun M.S, Dalsgaard I, Larsen L (2001). Incidence, distribution, and spread of tetracycline resistance determinants and integron-associated antibiotic resistance genes among motile aeromonads from a fish farming environment article a prendre comme exemple et comportant les amorces pour les in. Appl. Environ. Microbiol..

[40] Schmidt A.S, Bruun M.S, Larsen J.L, Dalsgaard I (2001). Characterization of class 1 integrons associated with R-plasmids in clinical *Aeromonas*
*salmonicida* isolates from various geographical areas. J. Antimicrob. Chemother..

[41] Aarestrup F.M, Agerso Y, Gerner-Smidt P, Madsen M, Jensen L.B (2000). Comparison of antimicrobial resistance phenotypes and resistance genes in *Enterococcus faecalis* and *Enterococcus faecium* from humans in the community, broilers, and pigs in Denmark. Diagn. Microbiol. Infect. Dis..

[42] Austin B, Austin D.A (2012). Bacterial Fish Pathogens:Disease of Farmed and Wild Fish.

[43] Shoits E.B, Rimler R (1973). Medium for the isolation of *Aeromonas hydrophila*. Appl. Microbiol.

[44] Peepim T, Dong H.T, Senapin S, Khunrae P, Rattanarojpong T (2016). Epr3 is a conserved immunogenic protein among *Aeromonas* species and able to induce antibody response in Nile tilapia. Aquaculture.

[45] Zhu M, Wang X.R, Li J, Li G.Y, Liu Z.P, Mo Z.L (2016). Identification and virulence properties of *Aeromonas veronii* bv. sobria isolates causing an ulcerative syndrome of loach *Misgurnus*
*anguillicaudatus*. J. Fish Dis..

[46] Radu S, Ahmad N, Ling F.H, Reezal A (2003). Prevalence and resistance to antibiotics for *Aeromonas* species from retail fish in Malaysia. Int. J. Food Microbiol..

[47] Kaskhedikar M, Chhabra D (2010). Multiple drug resistance in *Aeromonas hydrophila* isolates of fish. Vet. World.

[48] Ashiru A.W, Uaboi-Egbeni P.O, Oguntowo J.E, Idika C.N (2011). Isolation and antibiotic profile of *Aeromonas* species from Tilapia Fish (*Tilapia nilotica*) and Catfish (*Clarias batrachus*). Pak. J. Nutr..

[49] Esteve C, Biosca E, Amaro C (1993). Virulence of *Aeromonas hydrophila* and some other bacteria isolated from European eels *Anguilla anguilla* reared in fresh water. Dis. Aquat. Organ..

[50] Kumar S.P, Shankar R.S, Pasim R.K, Sabayasaohi P, Kumar D.N (2019). Pathogenic status, antibiogram, adhesive characteristics, heavy metal tolerance and incidence of integrons of infected fish isolated *Aeromonas* spp. J. Sustain. Sci. Manage..

[51] Cai S.H, Wu Z.H, Jian J.C, Lu Y.S, Tang J.F (2012). Characterization of pathogenic *Aeromonas*
*veronii* bv. veronii associated with ulcerative syndrome from Chinese longsnout catfish (*Leiocassis longirostris günther*). Braz. J. Microbiol..

[52] Sreedharan K, Philip R, Singh I.S.B (2011). Isolation and characterization of virulent *Aeromonas*
*veronii* from ascitic fluid of oscar *Astronotus*
*ocellatus* showing signs of infectious dropsy. Dis. Aquat. Organ..

[53] Kusdarwati R, Kismiyati, Sudarno, Kurniawan H, Prayogi Y.T (2017). Isolation and identification of *Aeromonas hydrophila* and *Saprolegnia* spp. on catfish (*Clarias gariepinus*) in floating cages in bozem moro Krembangan Surabaya. IOP Conf. Ser. Earth Environ. Sci.

[54] Nguyen H.N.K, Van T.T.H, Nguyen H.T, Smooker P.M, Shimeta J, Coloe P.J Molecular characterization of antibiotic resistance in *Pseudomonas* and *Aeromonas* isolates from catfish of the Mekong Delta, Vietnam. Vet. Microbiol..

[55] Odeyemi O.A, Ahmad A (2017). Antibiotic resistance profiling and phenotyping of *Aeromonas* species isolated from aquatic sources. Saudi J. Biol. Sci..

[56] Igbinosa I.H, Igumbor E.U, Aghdasi F, Tom M, Okoh A.I (2012). Emerging *Aeromonas* species infections and their significance in public health. Sci. World J.

[57] Tayler A.E, Ayala J.A, Niumsup P, Westphal K, Baker J.A, Zhang J.A, Zhang L, Walsh T.R, Wiedemann B, Bennett P.A, Avison M.B (2010). Induction of β-lactamase production in *Aeromonas hydrophila* is responsive to β-lactam-mediated changes in peptidoglycan composition. Microbiology.

[58] Janda J.M, Abbott S.L (2010). The genus *Aeromonas*:Taxonomy, pathogenicity, and infection. Clin. Microbiol. Rev..

[59] Cabello F.C, Godfrey H.P, Buschmann A.H, Dölz H.J (2016). Aquaculture as yet another environmental gateway to the development and globalisation of antimicrobial resistance. Lancet Infect. Dis.

[60] Dobiasova H, Kutilova I, Piackova V, Vesely T, Cizek A, Dolejska M (2014). Ornamental fish as a source of plasmid-mediated quinolone resistance genes and antibiotic resistance plasmids. Vet. Microbiol..

[61] National Pharmaceutical Regulatory Agency (2017). Ministry of Health Malaysia. List of Registered Veterinary Products. http://www.npra.moh.gov.my/images/reg.info/Veterinary/2017/LIST_OF_REGISTERED_VETERINARY_PRODUCTS_2017.pdf.

[62] European Food Safety Authority (2015). Scientific opinion on nitrofurans and their metabolites in food. EFSA J.

[63] Deng Y, Wu Y, Jiang L, Tan A, Zhang R, Luo L (2016). Multi-drug resistance mediated by class 1 integrons in *Aeromonas* isolated from farmed freshwater animals. Front. Microbiol..

[64] Dias C, Mota V, Martinez-Murcia A, Saavedra M.J (2012). Antimicrobial resistance patterns of *Aeromonas* spp. isolated from ornamental fish. J. Aquac. Res. Dev..

[65] Luo Y, Xu L, Rysz M, Wang Y, Zhang H, Alvarez P.J.J (2011). Occurrence and transport of tetracycline, sulfonamide, quinolone, and macrolide antibiotics in the Haihe River basin, China. Environ. Sci. Technol.

[66] Suzuki S, Hoa P.T.P (2012). Distribution of quinolones, sulfonamides, tetracyclines in aquatic environment and antibiotic resistance in Indochina. Front. Microbiol..

[67] Nawaz M, Khan S.A, Khan A.A, Sung K, Tran Q, Kerdahi K, Steele R (2010). Detection and characterization of virulence genes and integrons in *Aeromonas*
*veronii* isolated from catfish. Food Microbiol..

[68] Ndi O.L, Barton M.D (2011). Incidence of class 1 integron and other antibiotic resistance determinants in *Aeromonas* spp. From rainbow trout farms in Australia. J. Antimicrob. Chemother..

[69] Jones-Dias D, Manageiro V, Ferreira E, Louro D, Caniça M (2014). Diversity of extended-spectrum and plasmid-mediated AmpC β-lactamases in *Enterobacteriaceae* isolates from Portuguese health care facilities. J. Microbiol..

[70] Fosse T, Giraud-Morin C, Madinier I (2003). Phénotypes de résistance aux β-lactamines dans le genre *Aeromonas*. Pathol. Biol..

[71] Chen P.L, Lamy B, Ko W.C (2016). *Aeromonas**dhakensis*, an increasingly recognized human pathogen. Front. Microbiol..

[72] Vega-Sánchez V, Latif-Eugenin F, Soriano-Vargas E, Beaz-Hidalgo R, Figueras M.J, Aguilera-Arreola M.G, Castro-Escarpulli G (2014). Re-identification of *Aeromonas* isolates from rainbow trout and incidence of class 1 integron and β-lactamase genes. Vet. Microbiol..

